# Biomarkers of Good EULAR Response to the B Cell Depletion Therapy in All Seropositive Rheumatoid Arthritis Patients: Clues for the Pathogenesis

**DOI:** 10.1371/journal.pone.0040362

**Published:** 2012-07-30

**Authors:** Gianfranco Ferraccioli, Barbara Tolusso, Francesca Bobbio-Pallavicini, Elisa Gremese, Viviana Ravagnani, Maurizio Benucci, Edoardo Podestà, Fabiola Atzeni, Alice Mannocci, Domenico Biasi, Mariangela Manfredi, Piercarlo Sarzi-Puttini, Bruno Laganà, Carlomaurizio Montecucco

**Affiliations:** 1 Division of Rheumatology, Catholic University of the Sacred Heart, Rome, Italy; 2 Division of Rheumatology, University of Pavia, IRCCS S. Matteo Foundation, Pavia, Italy; 3 Department of Clinical and Experimental Medicine, Section of Rheumatology and Internal Medicine, University of Verona, Verona, Italy; 4 Rheumatology Unit, Department of Internal Medicine, Ospedale di S. Giovanni di Dio, Florence, Italy; 5 Division of Clinical Immunology and Rheumatology, II School of Medicine, S. Andrea University Hospital, “Sapienza” University of Rome, Rome, Italy; 6 Rheumatology Unit, University Hospital L. Sacco, Milan, Italy; 7 Unit of Hygiene, Department of Public Health and Infectious Diseases, Sapienza University of Rome, Rome, Italy; Innsbruck Medical University, Austria

## Abstract

**Objective:**

To find out whether a high number of auto-antibodies can increase the probability of a “good-EULAR response” and to identify the possible biomarkers of response in seropositive rheumatoid arthritis (RA) patients undergoing the B cell depletion therapy (BCDT).

**Patients and Methods:**

One hundred and thirty-eight patients with long standing RA (LSRA), 75% non or poorly responsive to one or more TNFα blockers, all seropositive for at least one autoantibody (AAB) (RF-IgM, RF-IgA, RF-IgG, anti-MCV, ACPA-IgG, ACPA-IgA, ACPA-IgM) received one full course of BCDT. The major outcomes (moderate or good-EULAR response) were assessed after 6 months of therapy. The IL6 and BAFF levels were also determined.

**Results:**

At a 6-month follow-up, 33 (23.9%) of the RA patients achieved a good EULAR response. Having up to 5-AABs positivity increased the chances for treatment response. After a logistic regression analysis, however, only 4 baseline factors arose as associated with a good-EULAR response: no steroid therapy (OR = 6.25), a lymphocyte count <1875/uL (OR = 10.74), a RF-IgG level >52.1 IU/ml (OR = 8.37) and BAFF levels <1011 pg/ml (OR = 7.38). When all the AABs, except for RF-IgM and ACPA-IgG, were left in the analysis, the two final predictors were no-steroid therapy and low lymphocyte count.

**Discussion:**

The number of AABs increased the chances of being a “good-EULAR” responder. The only predictors, however, at the baseline of a good response in this seropositive cohort of RA patients were 2 simple variables – no steroids and lymphocyte count – and two laboratory assays – IgG-RF and BAFF.

## Introduction

In rheumatoid arthritis (RA), the inflammation in the synovial tissue is an acute-chronic process that is caused by several inflammatory cells and cytokines [1]. Among the cellular players, the B cells are present in the synovial tissue of several types of arthritides where they act as an antigen presenting cells and producing cytokines and autoantibodies, some of which have been linked to a poor prognosis [2], [3]. Thus, we have been led to consider the B cells as a definite target in RA, especially the RA subtype with the poorest prognosis, i.e. the rheumatoid factor (RF) positive patients. The BCDT has become a possible second choice due to poor response to TNFα blockers in RA patients [4]–[6]. Data from clinical trials regarding BCDT indicate that patients positive for IgM rheumatoid factor (IgM-RF) and/or for anti-citrullinated peptide autoantibodies (ACPA) are those achieving the best clinical results in terms of ACR or good EULAR response [7], [8]. According to the data, the best candidate for the BCDT has been generally identified as the patient partially or fully refractory to a TNFα blocker, the RF or the ACPA positive. Therefore, seropositivity appears to be the best available biomarker in clinical practice for defining the best patient target for the treatment [9], [10]. Whether the IgM-RF or the ACPA, or both, are the best available biomarkers associated to treatment response still remains to be demonstrated.

Since not all seropositive patients obtain clinical benefits from the BCDT, one unfulfilled need is the identification of the ideal target among the autoantibody-positive patients, but no data are available regarding which parameters or variables can best identify the subject that benefits the most or the least among the seropositive RA patients. Furthermore, it is unknown whether multiple positivity of autoantibodies can confer a higher probability of response in RA.

In this study, we addressed the issue of which disease-specific characteristics, as well as biologic parameters, could be predictive factors of response to the BCDT in still active RA patients, in a cohort of patients all seropositive for at least one autoantibody (RF-IgG, IgA, or IgM, or ACPA-IgG, IgA or IgM or antibodies directed against a mutated citrullinated vimentin-anti-MCV). The primary aim was to define whether the higher the number of AABs, the higher would have been the chance of selecting the best responder. The secondary aim was to define biomarkers of good-EULAR response and possibly to obtain thresholds of each factor directly involved in the BCDT response that would be applicable in clinical practice.

## Patients and Methods

### Ethical statement

The ethical approval for the study was obtained from the Ethical Committee of the Catholic University of the Sacred Heart. All subjects gave their written informed consent.

### Patients

In this open-label retrospective, non-comparative, non-interventional study, RA patients from six Italian centers (two in Rome, Pavia, Florence, Verona, Milan), starting treatment with rituximab for the first time, were followed since 2006; thus, this is a retrospective observational study. Among the recruited patients, 138 AAB positive patients were included in the current investigation (see below). All patients had been diagnosed with RA following the American College of Rheumatology criteria [11] and admitted to the study because of an incomplete and/or poor response to conventional combination-DMARDs (Methotrexate-MTX, Leflunomide-LFN, Sulphasalazine-SSZ, Chloroquine-CHL) and/or TNFα blockers (one or more). They were still active after six months of combination therapy as either DMARDs or MTX+TNFα blockers. Steroids were allowed, but only to those patients who took the dosage of 5–7.5 mg/day and the patients that did not change the dosage over the study period were included in the analysis. The BCDT along with MTX (dose range: 10–25 mg/week according to tolerability) was used in 35 patients as the first choice due to comorbidities that excluded the anti-TNFα therapy. Eighteen patients, having had previous toxicities with MTX or other DMARDs, received the BCDT only. All patients received the BCDT (Rituximab 1 g twice, one infusion every two weeks). At baseline and every 3 months, demographic, clinical, including previous TNFα blocker therapies and current therapy with glucocorticoids, immunological and laboratory data, the Health Assessment Questionnaire (HAQ) and the disease activity score (DAS) were recorded. Clinical assessment using the DAS score was performed every three months of treatment. The EULAR response criteria based on the DAS were used to assess the disease activity during the follow-up. Good responders were defined subjects with a >1.2 improvement in the DAS from baseline and with a DAS attained during follow-up of ≤2.4. Non-responders were patients with an improvement of ≤0.6 or patients with an improvement of >0.6 but ≤1.2 and a DAS attained during follow-up of >3.7. Patients with a DAS ≤3.7 and an improvement of >0.6 were classified as moderate responders, that obviously include the good responders. [12].

Moreover, CD19 count, erythrocyte sedimentation rate (ESR) and C-reactive protein (CRP) and routine haematological and serum chemistry determinations were performed every three months. Serum immunoglobulin levels (IgG, IgA and IgM) were assessed by enzyme-linked immunosorbent assay (ELISA).

### Detection of Autoantibodies

IgA, IgG and IgM ACPA isotypes were measured using an EliA method on the ImmunoCAP 250 instrument (Phadia, Freiburg, Germany), with positive anti-CCP defined as ≥7 U/ml for IgG, ≥2.2 U/ml for IgA and ≥100 U/ml for IgM, as suggested by the manufacturer. The anti-MCV (anti-modified citrullinated vimentin) antibodies and the RF (IgG, IgA and IgM isotypes) were measured by ELISA (Orgentec Diagnostika GmbH, Mainz, Germany) and considered positive above a cut-off value of 20 U/ml as suggested by the manufacturer. The ACPAs and RFs were determined using plasma samples that had been stored. All the patients admitted to the study were positive for at least one autoantibody.

### Inflammatory biomarkers

Serum levels of IL-6 and BAFF were measured by ELISA (R&D Systems, UK). The sensitivity of the test was of 0.7 pg/ml for IL6 and 3.38 pg/ml for BAFF.

### Statistical analysis

A statistical analysis was performed using SPSS statistical software (SPSS for Macintosh, version 15 PSS, Chicago, IL, USA) and the MedCalc 8.0 package (MedCalc Software, Mariakerke, Belgium). Data were recorded as mean and Standard Deviation (SD) or number and percentage. In order to calculate the sample size, the following parameters were used: power 80%; level of confidence 95%; the frequency of “good response” to the BCDT after 6 months of treatment in the RA patients was 25.0% (based on the mean value of the European studies) [10] and the estimated frequency of 50.0%. The sample size calculation was performed using the formula for binary data. The sample size was estimated to be 130 RA cases.

A receiving operating curve (ROC) analysis [13] of the continuous parameters related to the “good EULAR response to the BCDT after the sixth month of follow-up” or “moderate EULAR response to the BCDT in the RA patients were performed in order to obtain relevant thresholds allowing the prediction of response to therapy at the individual level.

The nonparametric ROC plot uses all of the data, makes no parametric assumption and provides unbiased estimates of sensitivity and specificity. The calculation of area under the curve (AUC) provides a convenient single number. AUC values ≥0.550 or ≤0.450 were considered to be discriminative and 0.45<AUC<0.55 to be nondiscriminative. The optimal cutoff point was determined to yield the maximum corresponding sensitivity and specificity.

The following potential predictors of response to the BCDT therapy at a 6-month follow-up were evaluated using the χ^2^ test: gender; previous anti-TNF therapy; current steroid therapy and current DMARDs therapy; seropositivity for anti-MCV, ACPA (IgG, IgA or IgM), RF (IgG, IgA or IgM); baseline DAS score; baseline HAQ; baseline ESR, CRP, IL6 and BAFF levels and baseline lymphocytes count.

Two logistic multivariate regression models with stepwise backward-wald elimination to study the associations respect to two outcomes (good or moderate EULAR response) were used. The covariates included in the models had a p≤0.10 in the univariate analysis. The probability of stepwise to entry was settled at 0.05 and to remove was settled at 0.10. The goodness of fit of the models was performed using the Hosmer-Lemeshow test. Statistical significance was defined as *p<0.05*.

## Results

### Demographic, Clinical and Immunological Characteristics of the RA Patients at the baseline

The clinical, immunological and demographic characteristics at the baseline of the 138 RA patients are shown in Table 1. All patients had a moderate-active disease (DAS>2.4) as shown by the high DAS (4.5±1.2) and HAQ (1.67±0.75) scores. More specifically, 75% had a DAS score >3.7. In the RA cohort, 25.4% were TNF blockers naïve while 41.7%, 46.6% and 11.7% of the patients had failed one, two or three TNF inhibitors. One hundred and twenty-two patients were treated with MTX (range 10–20 mg), with the dosage remaining stable during the follow-up period. Overall 110 patients were receiving concomitant oral low-dose prednisone (range 5–7.5 mg/day). Eighteen of these patients were receiving only small doses of prednisone at the moment they underwent the BCDT, no MTX nor other DMARDs.

**Table 1:** Baseline demographic, clinical and immunological characteristics of RA patients included in the study.

	RA patients
**N**	**138**
**Gender: Female,** n. (%)	114 (82.6)
**Age** (years)	61.3±11.5
**Disease duration** (years)	13.0±10.6
**Anti-TNF naïve,** n (%)	35 (25.4)
**Previous anti-TNF agents used**	
**N = 1,** n (%)	43 (41.7)
**N = 2,** n (%)	48 (46.6)
**N = 3,** n (%)	12 (11.7)
**Current DMARDs therapy,** n (%)	122 (88.4)
**Current steroid therapy,** n (%)	110 (80.3)
**Tender joint count (n)**	18.8±12.6
**Swollen joint count (n)**	10.9±7.6
**CRP (mg/L)**	21.6±22.6
**ESR (mm/1^st^ hour)**	48.5±25.4
**DAS**	4.6±1.2
**HAQ**	1.66±0.74
**Anti-CCP IgG (U/ml)**	190.2±127.0 (151.0)*
**Anti-CCP IgG ≥7.0 U/ml,** n (%)	116/137 (84.7)
**Anti-CCP IgM (U/ml)**	371.1±281.1 (303.0)*
**Anti-CCP IgM ≥100 U/ml,** n (%)	33/137 (24.1)
**Anti-CCP IgA (U/ml)**	20.4±24.1 (7.5)*
**Anti-CCP IgA ≥2.2 U/ml,** n (%)	66/137 (48.2)
**RF-IgG (U/ml)**	174.0±216.7 (102.3)*
**RF-IgG ≥20U/ml,** n (%)	111/137 (81.0)
**RF-IgM (U/ml)**	156.3±158.3 (89.4)*
**RF-IgM ≥20U/ml,** n (%)	89 (64.5)
**RF-IgA (U/ml)**	159.5±176.0 (90.1)*
**RF-IgA ≥20U/ml,** n (%)	74 (53.6)
**Anti-MCV (U/ml)**	447.7±465.7 (216.3)*
**Anti-MCV ≥20U/ml,** n (%)	123 (89.1)

Values are mean ± sd unless otherwise indicated. *values are reported as mean ± sd (median).

RA = rheumatoid arthritis; TNF = tumor necrosis factor; DMARDs = disease modified anti-rheumatic drugs; CRP = C-reactive protein; ESR =  erythrocyte sedimentation rate; DAS =  disease activity score; HAQ = Health Assessment Questionnaire; CCP = cyclic citrullinated protein; RF =  rheumatoid factor; MCV =  modified citrullinated vimentin.

### Autoantibody Pattern in RA Patients Treated with BCDT

All the RA patients gave sera and were tested for all the autoantibody positivity, using the cut-off value recommended by the manufacturers. Among patients, 89 (64.5%) were RF-IgM positive, 116 (84.7%) were ACPA-IgG positive, 123 (89.1%) were positive for anti-MCV antibodies of which 113 (81.9%) were also anti-CCP (IgG) positive and 11 (8%) were only RF positive (IgG and/or IgM and/or IgA) (Table S1).

### EULAR Clinical Response after 6 Months of BCDT Treatment in Seropositive RA Patients

At end of the 6-month follow-up, 33 of the 138 (23.9%) RA patients achieved a good EULAR response while a moderate-EULAR response was reached in 71 (51.4%) patients and 34 (24.7%) RA patients were poor responders. Fourteen (10.1%) RA patients were in EULAR-remission at the end of the 6-month period.

Considering the cut-off levels recommended by the manufacturers for each autoantibody positivity, no association was seen between the AAB pattern and the response to the BCDT therapy. (Table S1). The RA patients reaching a good-EULAR response to BCDT at the sixth month follow-up had a significantly different distribution of the number of AAB positivity compared to the patients with a poor-EULAR response (χ^2^ test = 16.69, *p = 0.01*). In particular, the RA patients reaching the good-EULAR response had 3 to 5 AAB positivity in much higher percentage (84.8%) that poor-EULAR responders (46.7%, *p<0.001*). Moreover, the cut-off number resulted from the ROC curve analysis (AUC = 0.536±0.051) for the positivity of AAB was 5 (Table 2 and Figure S1).

**Table 2:** ROC curve analysis: Area Under the Curve (AUC) for considered biomarkers in good or moderate-EULAR responders compared to poor-EULAR responder RA patients.

Variable	Good-EULAR response			Moderate-EULAR response*		
	AUC	Std.Error^a^	*p^b^*	AUC	Std.Error^a^	*p^b^*
HAQ	0.639	0.068	*0.05*	0.242	0.061	*0.001*
Lymphocytes (u/ul)	0.655	0.072	*0.03*	0.297	0.067	*0.01*
ESR (mm/1^st^ hr)	0.601	0.072	*0.16*	0.383	0.073	*0.12*
CRP (mg/l)	0.623	0.072	*0.09*	0.377	0.072	*0.11*
BAFF (pg/ml)	0.633	0.066	*0.09*	0.402	0.076	*0.20*
IL6 (pg/ml)	0.621	0.069	*0.07*	0.380	0.075	*0.12*
Anti-CCP IgG (U/ml)	0.486	0.061	*0.61*	0.381	0.077	*0.12*
Anti-CCP IgM (U/ml)	0.482	0.064	*0.78*	0.515	0.078	*0.84*
Anti-CCP IgA (U/ml)	0.518	0.062	*0.78*	0.437	0.078	*0.41*
RF-IgG (U/ml)	0.379	0.062	*0.07*	0.546	0.077	*0.55*
RF-IgM (U/ml)	0.439	0.065	*0.36*	0.544	0.078	*0.56*
RF-IgA (U/ml)	0.555	0.067	*0.50*	0.451	0.076	*0.52*
Anti-MCV (U/ml)	0.434	0.060	*0.49*	0.418	0.078	*0.28*
AB positivity	0.535	0.051	*0.54*	0.456	0.079	*0.56*

a Under the nonparametric assumption; ^b^Null hypothesis: true area  = 0.5.

* Subjects included in the group “moderate-EULAR response” were patients that have reached good or moderate response. CRP = C-reactive protein; ESR =  erythrocyte sedimentation rate; HAQ = Health Assessment Questionnaire; BAFF =  B cell activating factor; IL =  interleukin; CCP = cyclic citrullinated protein; RF =  rheumatoid factor; MCV =  modified citrullinated vimentin; AB =  autoantibodies.

As shown in Table S2, the variables related to response to the BCDT therapy at the sixth month follow-up in univariate analysis were no current steroid therapy (*p<0.001*), a DAS score of less than 3.7 and a HAQ score less than 1.5 (*p = 0.01*), a baseline lymphocyte count <1875/μl (*p = 0.002*), a CRP<5 mg/l (*p = 0.01*), BAFF<1011 pg/ml (*p = 0.04*) and IL6<15 pg/ml (*p = 0.01*) levels and a baseline anti-MCV>36.5 U/ml (*p = 0.04*) and RF-IgG>52.1 U/ml (*p = 0.01*) AAB levels.

In the logistic regression analysis, the best independent predictors of “good EULAR response to the BCDT after the sixth month follow-up” in the RA patients were baseline lymphocyte count <1875/uL (OR (95% CI): 10.74 (2.21–52.13)), RF IgG levels >52.1 IU/ml (OR (95% CI): 8.37 (1.34–52.14)), plasma BAFF levels <1011 pg/ml (OR (95% CI): 7.38 (1.24–43.76)) and no-current steroid therapy (OR (95% CI): 6.25 (1.28–33.33)) (Table 3). The IgG, IgA and IgM levels, as well as the titres of the AABs, resulted not significant.

**Table 3:** Logistic regression model t predicting 6^th^ month good- EULAR response to BCDT in RA patients.

Variables	OR (95% CIs)
Current steroid therapy yes = 1	**0.16 (0.03–0.78)**
DAS, <3.7 = 1	0.88 (0.15–5.06)
HAQ, <1.5 = 1	2.24 (0.49–10.18)
Lymphocytes, <1875/ul = 1	**10.74 (2.21–52.13)**
ESR, <30mm/1st hr = 1	5.55 (0.93–29.00)
CRP, <5mg/l = 1	2.17 (0.21–21.95)
BAFF, <1011 pg/ml = 1	**7.38 (1.24–43.76)**
IL6, <15 pg/ml = 1	3.59 (0.78–16.40)
IgG-RF, >52.1U/ml = 1	**8.37 (1.34–52.14)**
IgA-RF, <37U/ml = 1	3.34 (0.68–16.45)
Anti-MCV, >36.5U/ml = 1	6.17 (0.63–60.07)
*Hosmer and Lemeshow test*	*p = 0.95*

The cut-off values for continuous variables related to the “good EULAR response to BCDT after 6^th^ months FU” were obtained with ROC curves analysis. OR = odds ratio; 95%CI = 95% confidence interval; CRP = C-reactive protein; ESR =  erythrocyte sedimentation rate; DAS =  disease activity score; HAQ = Health Assessment Questionnaire; CCP = cyclic citrullinated protein; RF =  rheumatoid factor; MCV =  modified citrullinated vimentin. Boldface type indicates that *P* value is less than *0.05*.

Considering the number of autoantibody positivity instead of dichotomous single AAB values, we have again found that the best independent predictors of “good EULAR response to the BCDT after the sixth month FU” in RA patients were the baseline low lymphocyte count <1875/uL (OR (95% CI): 7.45 (1.96–28.27)), plasma BAFF levels <1011 pg/ml (OR (95% CI): 7.16 (1.43–35.74)) and no current steroid therapy (OR (95% CI): 11.23 (2.56–50.00)). In the last step of the model, the number of AAB positive up to 5 remained, even though it did not reach statistical significance (OR (95% CI): 4.50 (0.95–26.21)).

Using the model shown in Table 3 for the outcome, but without the contribution of the IgG-RF parameter, we have shown that the best independent predictors of “good EULAR response to the BCDT after the sixth month FU” in the RA patients were a baseline lymphocyte count of <1875/uL (OR (95% CI): 7.92 (2.08–30.23)), plasma BAFF levels <1011 pg/ml (OR (95% CI): 7.58 (1.48–38.90)) and no current steroid therapy (OR (95% CI): 11.11 (2.50–50.00)). The use of the same model reported in Table 3, together with the removal of all the AAB, except for the IgM-RF and ACPA-IgG in the stepwise multivariate regression analysis revealed both a low lymphocyte count and no-steroids as the best predictors.

**Figure 1:**
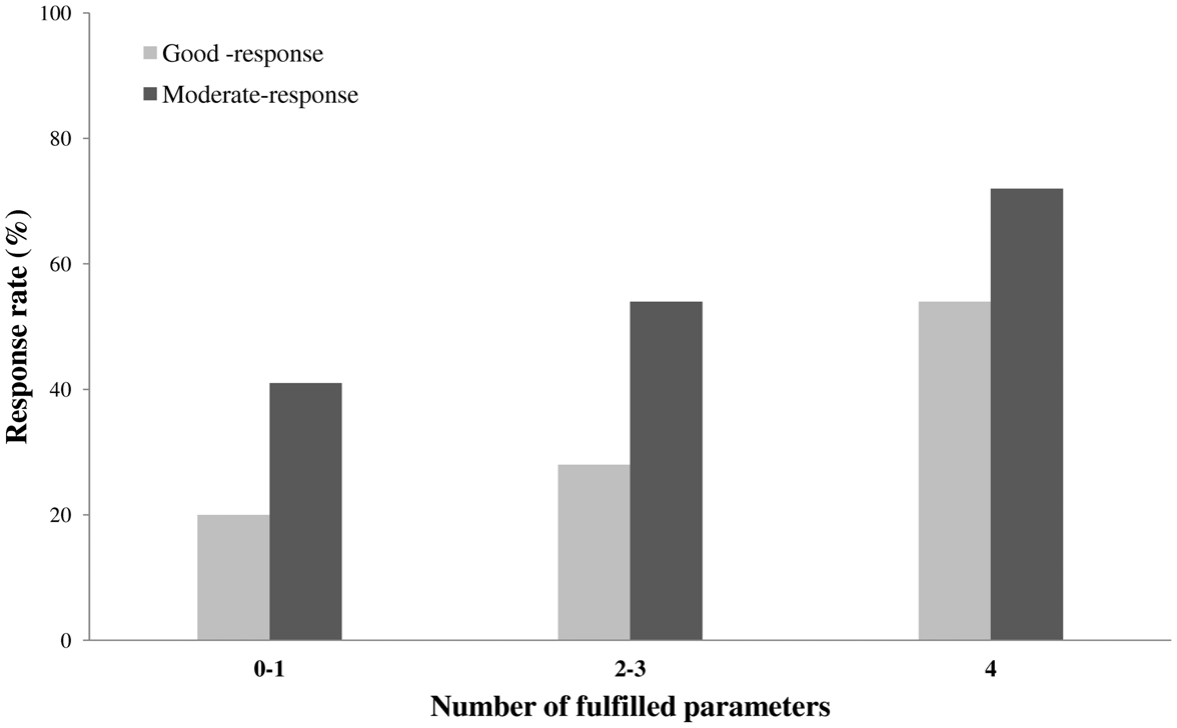
Percentage of good and moderate EULAR response rate (%) in RA patients after 6 months of RTX therapy. Patients were grouped according to the number of fulfilled parameters (corticosteroids therapy, number of circulating lymphocytes <1875/μl, plasma BAFF levels <1011 pg/ml and RF-IgM >52.1 U/ml). Good response was reached in 20.8% of RA patients that fulfilled 0–1 parameter, in 29.2% of subjects that fulfilled 2–3 parameters and in 54.5% of subjects that fulfilled 4 parameters (Fisher's exact test between 4 parameters and 0–3 = *0.08*). Moderate-response was reached in 41.7% of RA patients that fulfilled 0–1 parameter, in 54.5% of subjects that fulfilled 2–3 parameters and in 72.7% of subjects that fulfilled 4 parameters (χ2 test = 3.02, df = 2; *p = 0.22*). *p = 0.046: percentage of good response in patients fulfilling 4 *vs* 0–1 parameters.

After considering the contribution of the 4 predictive parameters, lymphocyte count, the BAFF levels, no current steroid therapy, IgG-RF autoantibody level, we observed that a higher percentage of patients fulfilling all the parameters obtained a good-response compared to patients satisfying none or only one parameter (54.5% vs 20.8%, *p = 0.046*). (Figure 1).

In order to see whether the “good-EULAR response” was a too strict clinical endpoint and then it could identify too selective biomarkers, we analyzed all the data in light of the “moderate EULAR response”. The variables emerging from the univariate analysis showed that a lymphocyte count<1546 μL and no-current steroid therapy again arose as the most significant among all the variables (Table S3).

At the logistic regression analysis, the baseline lymphocyte count <1546 μL (OR (95% CI): 278.62 (8.71–8911.03)), plasma BAFF levels <1002 pg/ml (OR (95% CI): 25.93 (1.95–344.19)) and no-current steroid therapy (OR (95% CI): 20.0 (1.00–500.00)) emerged again as significant independent factors among the best independent predictors of “moderate EULAR response” to the BCDT after the 6-month FU (Table 4). In addition a low HAQ and a positive anti-MCV arose as co-factors predicting a moderate response.

**Table 4:** Logistic regression model to predicting 6^th^ month moderate EULAR response to BCDT in RA patients.

Variables	OR (95% CI)
Current steroid therapy yes = 1	**0.05 (0.002–0.98)**
DAS, <3.7 = 1	2.59 (0.32–20.92)
HAQ, <1.0 = 1	**48.33 (2.78–840.24)**
Lymphocytes, <1546/ul = 1	**278.62 (8.71–8911.03)**
ESR, <60mm/1st hr = 1	5.38 (0.56–51.59)
CRP, <5mg/l = 1	0.33 (0.01–9.48)
BAFF, <1002 pg/ml = 1	**25.93 (1.95–344.19)**
IL6, <20.2 pg/ml = 1	3.04 (0.32–28.79)
IgG-ACPA, <273U/ml = 1	1.56 (0.02–117.97)
IgM-ACPA, <158U/ml = 1	3.79 (0.05–276.59)
IgG-RF, >25.6U/ml = 1	0.81 (0.04–15.91)
IgM-RF, >38.6U/ml = 1	**19.93 (2.21–180.22)**
Anti-MCV, <407.7U/ml = 1	**56.29 (1.71–1847.17)**
*Hosmer and Lemeshow test*	*p = 0.63*

The cut-off values for continuous variables related to the “moderate-EULAR response to BCDT after 6^th^ months FU” were obtained with ROC curves analysis. OR = odds ratio; 95%CI = 95% confidence interval; CRP = C-reactive protein; ESR =  erythrocyte sedimentation rate; DAS =  disease activity score; HAQ = Health Assessment Questionnaire; CCP = cyclic citrullinated protein; RF =  rheumatoid factor; MCV =  modified citrullinated vimentin. Boldface type indicates that P value is less than 0.05.

## Discussion

Optimizing therapy in RA becomes more and more important given the huge cost of all biologics once RA does not respond to methotrexate [14] or to combination therapy with conventional DMARDs [15], [16]. On the one hand, a way to optimize the therapy is the choice of biologics on the basis of the best responders or, on the other hand, of the worst responder. Among the biologics, the BCDT with rituximab has become a standard of therapy in poor responder patients to TNFα blockers. Some investigators suggest using it after the first TNFα blocker failure [17], [18]. Thus, identifying the best possible responder is of fundamental importance. In fact, a delay of at least 4 to 6 months is needed before drawing an initial conclusion to the response to the BCDT and 6 months can cause structural damage if the disease activity does not decrease. In the case of a poor clinical control this might lead to sustained structural damage [19].

No real clues have been detected from tissues. In some studies, an attempt was made to use the immunohistological analysis of the synovial membrane as a way to identify the best response. No baseline data emerged as possible response biomarkers, but the better the depletion over time the better the clinical results. Moreover, the study from Teng et al demonstrated that a low disease activity following rituximab treatment was associated with reduced infiltration of early plasma cells in synovium, suggesting that the reduction of disease activity by BCDT in patients with RA could be explained by the presence of VD79a+ plasma cells in the synovium [20]–[22].

Trial data suggested that IgM-RF could be used to identify the best target [23]. In the Serene trial, the analysis of predictors led to identify all isotypes of RF as well as IgG-ACPA as possible predictors of a good response [24]. Even preliminary data in a retrospective cohort, from randomly chosen patients confirmed RF as a possible biomarker [9]. On the other hand, another larger observational cohort showed that ACPA-IgG were a better biomarker of good-EULAR response than IgM-RF [10]. The rationale that both AABs could be important was supported by the French multicenter study carried out on 208 patients in which 21% reached a good-EULAR and 50% a partial-EULAR response after BCDT therapy. It showed that the response was associated with IgM-RF or ACPA or high IgG levels, thus suggesting that the 3 variables could allow for the selection of the best responder before treatment [25]. Note that 50% of the seronegatives, either for ACPA-IgG or for IgM-RF, responded. Moreover, in the French cohort the majority of the patients were anti-TNF poor responders. Therefore, the biomarkers can only be applied to the TNF-blockers poor responder patients. All data derived from pooling seropositive and seronegative patients cannot be applied to fully seropositive patients. But the data seem to confirm that both AAB are crucial and the double seropositivity could involve a higher risk.

In a small retrospective cohort, a BLyS single nucleotide polymorphism (SNPs), which is generally associated with low levels of the cytokine, appeared as a possible biomarker of moderate-good response [25], but data have yet to be confirmed.

To answer some of the questions, we studied only the seropositive RA patients. The selection of all seropositive patients was made with the idea that, since seropositivity seems to be the main driver, then the higher the positivity of the isoform of RF or ACPA, the higher should have been the chance of selecting the best responder. In our cohort, we observed that the higher the number of AAB the higher the chance of getting a response to BCDT. In addiction, we were able to identify, in the logistic regression analysis, 4 parameters which are associated strictly to a good-EULAR response. Two of these biomarkers are easily identifiable at the baseline, i.e. a low lymphocyte count and no-steroid therapy and two others are biological (high IgG-RF and low BAFF plasma levels). Neither gender, nor previous therapies, arose as predictors, even though we observed more than a 2-fold higher chance of obtaining remission in patients not previously treated with TNF- blockers.

As mentioned in the French cohort of patients with long-standing RA, anti-CCP or RF-IgM positivity and serum levels of IgG >1266 mg/dl (upper normal limit) arose as predictors of EULAR response. The good-EULAR response was reached in 22% of the patients [25]. Considering the moderate response our data confirm the importance of RF-IgM, and suggest that anti-MCV (not ACPA) could be predictors. Therefore, we only partially confirmed the results of this study in that we have not found an association between levels of auto-antibodies and EULAR response. We observed some relationship with a higher number of AABs. In our cohort, patients having RF without anti-CCP were very few, thus explaining why a possible additive effect could hardly have been identified. Sellam's study [26] did not reveal any useful information from the free light chain assessment, nor from BAFF levels. In our study the baseline lymphocyte count, the plasma BAFF levels, along with no-corticosteroid therapy arose as strong predictors of the response. On the other hand, Lai et al observed that all AABs (RF of all isotypes as well as IgG-ACPA) were associated with a placebo-corrected ACR50 response, thus suggesting that being seropositive, irrespectively of the AAB, led to higher clinical response [24].

Certainly, our cohort differs from the French one in that the number of ACPA-IgG and IgM-RF was lower in our study. The number of patients treated with steroids was a bit higher. The number of TNF-blocker untreated patients was certainly higher in our study than in the French cohort. Most importantly, all our patients were seropositive for at least one AAB. In our view, our results obtained in a cohort of previously treated, but all seropositive RA patients shed more light on the scientific issue and offer the opportunity to speculate on several biological questions. First, not using steroids in patients with active disease means no compartmentalization nor lymphocytolysis before using the BCDT, a very significant consideration when dealing with a drug (Rituximab) that acts, among several mechanisms, through the lysis, a complement dependent, antibody dependent cellular cytotoxicity of the B cells. Our findings also mean that patients with low lymphocyte counts can have an intrinsically favorable prognosis. Secondly, the presence of high IgG-RF and low levels of BAFF suggest a specific autoimmune inflammatory milieu. IgG-RF has long been considered the main driver of autoimmune rheumatoid inflammation, since small size immune complexes containing IgG-RF appear to be consistently and continuously monocyte-activating, contributing to the accrued local production of TNFα and IL1 [27]. The data confirm Edward's original hypothesis regarding the consistent role of IgG-RF in driving the persistence of RA inflammation [28] and the demonstration is that, by depleting the B cells, along with a clinical improvement, a drop of IgG-RF occurs [29]. Thus, there is indirect evidence that the BCDT could be one mechanism of progressive de-activation of rheumatoid inflammation. The other interesting point observed in our analysis is the predictive power of low plasma levels of BAFF at the baseline. It is well recognized that BAFF plays a crucial role in the development and survival of B cells [30]. We previously observed that clinical improvement in early RA associated with a drop in the BAFF plasma levels suggests that though not strictly linked to disease activity, BAFF represents one of the molecules that drives the persistence of rheumatoid inflammation [31]. Low BAFF levels are associated to a lower B cell survival and thus, likely allow a better biological effect of the BCDT. Of course, were this the case, other strategies could be envisioned in order to have lower levels of the BAFF, before initiating the BCDT in RA. Certainly, the low BAFF as a prognostic factor of a good response to BCDT raises a lot of interesting questions about how to modulate the environment of RA synovial inflammation in order to put into practice a personalized treatment and to reach a sustained remission [32]–[34].

## Supporting Information

Figure S1 **The ROC analyses were used to evaluate the cut-off of the considered biomarkers as predictors of a good-EULAR response (A–B) and moderate-EULAR response (C–D).**
(TIF)

Table S1 **Autoantibodies distribution in RA patients receiving rituximab therapy.**
(DOC)

Table S2 **Univariate analysis of dichotomous baseline clinical and laboratory parameters associated with “6 months good-EULAR response to BCDT” in RA patients.**
(DOC)

Table S3 Univariate analysis of dichotomous baseline clinical and laboratory parameters associated with 6 months moderate-EULAR response* to BCDT in RA patients.
(DOC)
