# Supplementary figures and images for: Biomarkers of Good EULAR Response to the B Cell Depletion Therapy in All Seropositive Rheumatoid Arthritis Patients: Clues for the Pathogenesis
Source: PLoS One. 2012 Jul 30;7(7):e40362. doi: 10.1371/journal.pone.0040362 (PMC3408482; doi:10.1371/journal.pone.0040362)

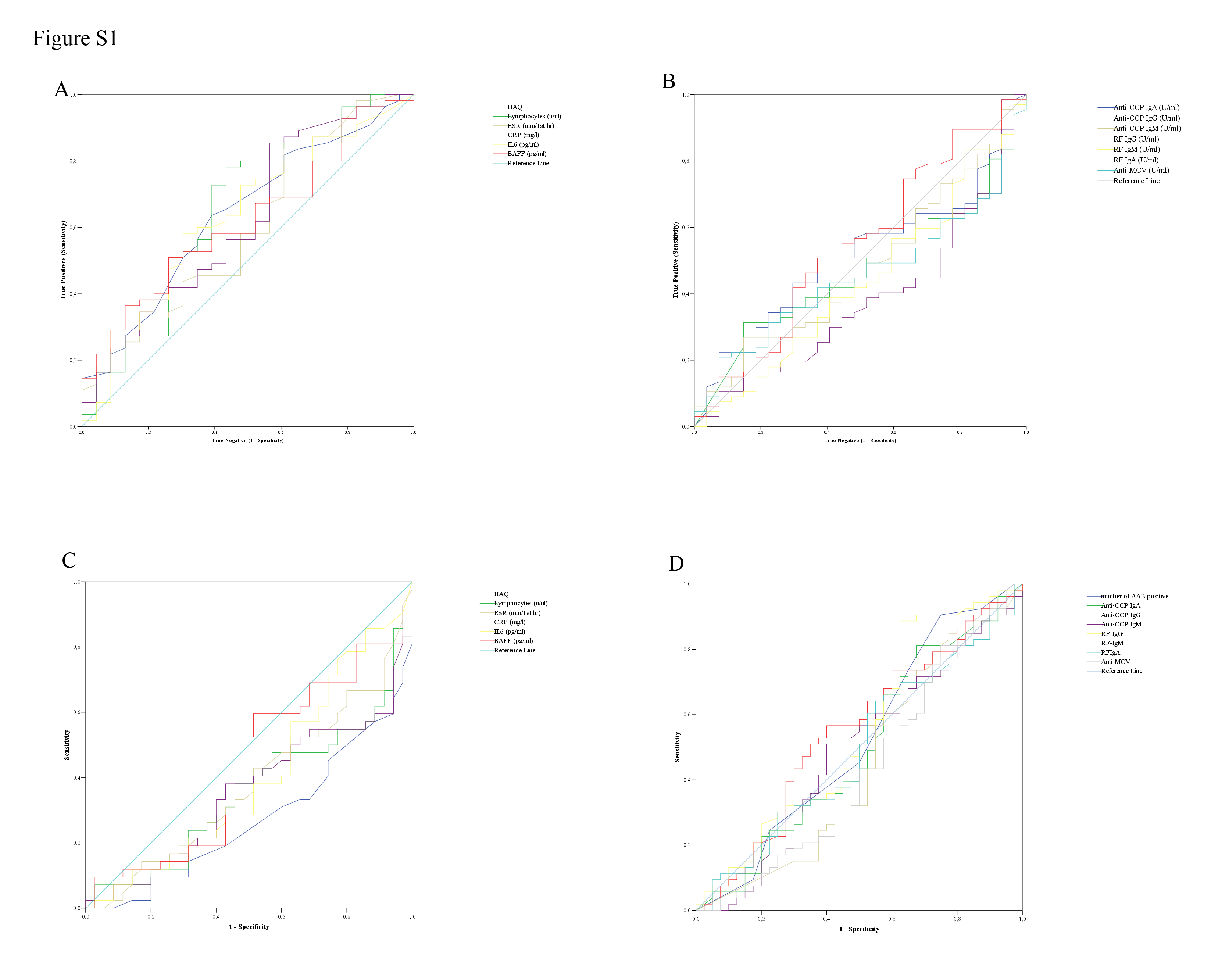

Supplement: Figure S1 — The ROC analyses were used to evaluate the cut-off of the considered biomarkers as predictors of a good-EULAR response (A–B) and moderate-EULAR response (C–D). (TIF) [file pone.0040362.s001.tif]
