# Supplementary material for: Biomarkers of Good EULAR Response to the B Cell Depletion Therapy in All Seropositive Rheumatoid Arthritis Patients: Clues for the Pathogenesis
Source: PLoS One. 2012 Jul 30;7(7):e40362. doi: 10.1371/journal.pone.0040362 (PMC3408482; doi:10.1371/journal.pone.0040362)
Supplement: Table S1 — Autoantibodies distribution in RA patients receiving rituximab therapy. (DOC) [file pone.0040362.s002.doc]

**Table S1.**

|  | **RA**  **GENERAL COHORT** | **RA**  **GOOD**  **RESPONDERS**  **(T6)** | **RA**  **POOR**  **RESPONDERS**  **(T6)** | ***p*** |
| --- | --- | --- | --- | --- |
| **N** | 138 | 33 | 105 |  |
| **Anti-CCP IgG ≥ 7.0 U/ml,** n (%) | 117 (84.8) | 30 (90.9) | 87 (82.9) | *0.26* |
| **Anti-CCP IgM ≥ 100 U/ml,** n (%) | 33 (23.9) | 4 (12.1) | 29 (27.6) | *0.10* |
| **Anti-CCP IgA ≥ 2.2 U/ml,** n (%) | 67 (48.6) | 12 (36.4) | 55 (52.4) | *0.11* |
| **RF-IgG ≥ 20U/ml,** n (%) | 111 (80.4) | 30 (90.9) | 81 (77.1) | *0.13* |
| **RF-IgM ≥ 20U/ml,** n (%) | 90 (65.2) | 22 (66.7) | 68 (64.8) | *0.84* |
| **RF-IgA ≥ 20U/ml,** n (%) | 74 (53.6) | 14 (42.4) | 60 (57.1) | *0.14* |
| **Anti-MCV ≥ 20U/ml,** n (%) | 123 (89.1) | 32 (97.0) | 91 (86.7) | *0.12* |
| **Anti-MCV+ and Anti-CCP IgG+,** n (%) | 113 (81.9) | 30 (90.9) | 83 (79.0) | *0.19* |
| **Anti-MCV+ and RF-IgM+,** n (%) | 81 (58.7) | 22 (66.7) | 59 (56.2) | *0.34* |
| **Anti-CCP IgG+ and RF-IgM+,** n (%) | 78 (56.5) | 20 (60.6) | 58 (55.2) | *0.59* |
| **RF alone (IgG and/or IgM and/or IgA),**  n (%) | 11 (8.0) | 1 (3.0) | 10 (9.5) | *0.46* |
| **N° of AB+,** mean ± sd | 4.45 ± 1.75 | 4.36 ± 1.27 | 4.48 ± 1.88 | *0.46* |
| **AB+>5,** n (%) | 43 (31.2) | 4 (12.1) | 39 (37.1) | ***0.01*** |

RA=rheumatoid arthritis; CCP=cyclic citrullinated protein; RF= rheumatoid factor; MCV= modified citrullinated vimentin; AB: autoantibodies.
