# Supplementary material for: Biomarkers of Good EULAR Response to the B Cell Depletion Therapy in All Seropositive Rheumatoid Arthritis Patients: Clues for the Pathogenesis
Source: PLoS One. 2012 Jul 30;7(7):e40362. doi: 10.1371/journal.pone.0040362 (PMC3408482; doi:10.1371/journal.pone.0040362)
Supplement: Table S2 — Univariate analysis of dichotomous baseline clinical and laboratory parameters associated with “6 months good-EULAR response to BCDT” in RA patients. (DOC) [file pone.0040362.s003.doc]

**Table S2.**

| **Variables** | **RA**  **Good Responders (T6)**  **N=33** | **RA**  **Poor Responders (T6)**  **N=105** | **OR (95% CI)** | ***p*** |
| --- | --- | --- | --- | --- |
| Sex, Female=1 | 27 (81.8) | 87 (82.9) | 0.93 (0.34-2.58) | *0.89* |
| Anti-TNF therapy, yes=1 | 21 (63.6) | 82 (78.1) | 0.49 (0.21-1.15) | *0.11* |
| Current steroid therapy yes=1 | 19 (57.6) | 90/104 (86.5) | **0.21 (0.09-0.52)** | ***<0.001*** |
| DMARDs therapy, yes=1 | 27 (81.8) | 95 (90.5) | 0.47 (0.16-1.42) | *0.18* |
| DAS, < 3.7 = 1 | 11/27 (40.7) | 16/92 (17.4) | **3.30 (1.30-8.30)** | ***0.01*** |
| HAQ, < 1.5 =1 | 19/30 (63.3) | 37/104 (35.6) | **3.13 (1.34-7.28)** | ***0.01*** |
| Lymphocytes, <1875/ul =1 | 16/26 (61.5) | 18/66 (27.3) | **4.27 (1.64-11.12)** | ***0.002*** |
| ESR, <30mm/1st hr =1 | 12 (36.4) | 21 (20.0) | 2.29 (0.90-5.40) | *0.06* |
| CRP, <5mg/l = 1 | 11/32 (34.4) | 15 (14.3) | **2.90 (1.18-7.20)** | ***0.01*** |
| BAFF, < 1011 pg/ml =1 | 25 (75.8) | 43/78 (55.1) | **2.50 (1.02-6.30)** | ***0.04*** |
| IL6, <15 pg/ml =1 | 23/31 (74.2) | 37/82 (45.1) | **3.50 (1.40-8.70)** | ***0.01*** |
| IgG-ACPA, >28U/ml =1 | 25/29 (86.2) | 54/79 (68.4) | 2.90 (0.90-9.20) | *0.09* |
| IgA-ACPA, <2.2U/ml = 1 | 18/29 (62.1) | 36/78 (46.2) | 1.90 (0.80-4.60) | *0.14* |
| IgM-ACPA, <100U/ml =1 | 25/29 (86.2) | 56/79 (70.9) | 2.56 (0.80-8.20) | *0.14* |
| IgG-RF, >52.1U/ml = 1 | 23/30 (76.7) | 44/90 (48.9) | **3.44 (1.34-8.81)** | ***0.01*** |
| IgM-RF, >20U/ml =1 | 21/32 (65.6) | 64/101 (63.4) | 1.10 (0.48-2.54) | *0.82* |
| IgA-RF, <37U/ml =1 | 23/32 (71.9) | 54/101 (53.5) | 2.22 (0.94-5.30) | *0.07* |
| Anti-MCV, >36.5U/ml =1 | 29/31 (93.5) | 73/96 (76.0) | **4.60 (1.01-20.6)** | ***0.04*** |

The cut-off values for continuous variables related to the “good- EULAR response to BCDT after 6^th^ months FU” were obtained with ROC curves analysis. OR=odds ratio; 95%CI=95% confidence interval; TNF=tumor necrosis factor; DMARDs=disease modified anti-rheumatic drugs; CRP=C-reactive protein; ESR= erythrocyte sedimentation rate; DAS= disease activity score; HAQ=Health Assessment Questionnaire; CCP=cyclic citrullinated protein; RF= rheumatoid factor; MCV= modified citrullinated vimentin.
