# Supplementary material for: Biomarkers of Good EULAR Response to the B Cell Depletion Therapy in All Seropositive Rheumatoid Arthritis Patients: Clues for the Pathogenesis
Source: PLoS One. 2012 Jul 30;7(7):e40362. doi: 10.1371/journal.pone.0040362 (PMC3408482; doi:10.1371/journal.pone.0040362)
Supplement: Table S3 — Univariate analysis of dichotomous baseline clinical and laboratory parameters associated with 6 months moderate-EULAR response* to BCDT in RA patients. (DOC) [file pone.0040362.s004.doc]

**Table S3.**

| **Variables** | **OR (95% CI)** |
| --- | --- |
| Sex, Female=1 | 0.87 (0.36-2.12) |
| Anti-TNF therapy, yes=1 | 0.74 (0.34-1.59) |
| Current steroid therapy yes=1 | **0.35 (0.14-0.87)** |
| DMARDs therapy, yes=1 | 1.42 (0.50-4.05) |
| DAS, < 3.7 = 1 | **5.98 (2.08-17.18)** |
| HAQ, < 1.0 =1 | **4.03 (1.58-10.26)** |
| Lymphocytes, <1546/ul =1 | **11.53 (2.49-53.38)** |
| ESR, <60mm/1st hr =1 | **2.93 (1.32-6.47)** |
| CRP, <5mg/l = 1 | **3.43 (1.34-8.77)** |
| BAFF, < 1002 pg/ml =1 | **3.08 (1.40-6.77)** |
| IL6, <20.2 pg/ml =1 | **3.01 (1.40-6.76)** |
| IgG-ACPA, <273U/ml =1 | **2.86 (1.26-6.71)** |
| IgA-ACPA, <2.2U/ml = 1 | 1.51 (0.70-3.26) |
| IgM-ACPA, <158U/ml =1 | **3.34 (1.16-9.62)** |
| IgG-RF, >25.6U/ml = 1 | **2.92 (1.15-7.43)** |
| IgM-RF, >38.6U/ml =1 | 1.57 (0.79-3.13) |
| IgA-RF, <37U/ml =1 | 1.38 (0.69-2.76) |
| Anti-MCV, <407.7U/ml =1 | **2.48 (1.17-5.28)** |

The cut-off values for continuous variables related to the “moderate-EULAR response to BCDT after 6^th^ months FU” were obtained with ROC curves analysis. OR=odds ratio; 95%CI=95% confidence interval; TNF=tumor necrosis factor; DMARDs=disease modified anti-rheumatic drugs; CRP=C-reactive protein; ESR= erythrocyte sedimentation rate; DAS= disease activity score; HAQ=Health Assessment Questionnaire; CCP=cyclic citrullinated protein; RF= rheumatoid factor; MCV= modified citrullinated vimentin. *Subjects included in the group “moderate-EULAR response” were patients that have reached good or moderate response.
